# Identification of de novo mutations for *ARID1B* haploinsufficiency associated with Coffin–Siris syndrome 1 in three Chinese families via array-CGH and whole exome sequencing

**DOI:** 10.1186/s12920-021-01119-2

**Published:** 2021-11-14

**Authors:** Guanting Lu, Qiongling Peng, Lianying Wu, Jian Zhang, Liya Ma

**Affiliations:** 1 Department of Pathology, Laboratory of Translational Medicine Research, Deyang Key Laboratory of Tumor Molecular Research, Deyang People’s Hospital, No. 173 First Section of TaishanBei Road, Jiangyang District, Deyang, 618000 China; 2 Department of Child Healthcare, Shenzhen Baoan Women’s and Children’s Hospital, Jinan University, 56 Yulyu Road, Baoan District, Shenzhen, 518000 China

**Keywords:** Haploinsufficiency, ARID1B, Coffin–Siris syndrome, SWI/SNF complex, Microdeletion, Loss-of-function

## Abstract

**Background:**

Coffin–Siris syndrome (CSS) is a multiple malformation syndrome characterized by intellectual disability associated with coarse facial features, hirsutism, sparse scalp hair, and hypoplastic or absent fifth fingernails or toenails. CSS represents a small group of intellectual disability, and could be caused by at least twelve genes. The genetic background is quite heterogenous, making it difficult for clinicians and genetic consultors to pinpoint the exact disease types.

**Methods:**

Array-Comparative Genomic Hybridization (array-CGH) and whole exome sequencing (WES) were applied for three trios affected with intellectual disability and clinical features similar with those of Coffin–Siris syndrome. Sanger sequencing was used to verify the detected single-nucleotide variants (SNVs).

**Results:**

All of the three cases were female with normal karyotypes of 46, XX, born of healthy, non-consanguineous parents. A 6q25 microdeletion (arr[hg19]6q25.3(155,966,487–158,803,979) × 1) (2.84 Mb) (case 1) and two loss-of-function (LoF) mutations of *ARID1B* [c.2332 + 1G > A in case 2 and c.4741C > T (p.Q1581X) in case 3] were identified. All of the three pathogenic abnormalities were de novo, not inherited from their parents. After comparison of publicly available microdeletions containing *ARID1B*, four types of microdeletions leading to insufficient production of *ARID1B* were identified, namely deletions covering the whole region of *ARID1B*, deletions covering the promoter region, deletions covering the termination region or deletions covering enhancer regions.

**Conclusion:**

Here we identified de novo ARID1B mutations in three Chinese trios. Four types of microdeletions covering ARID1B were identified. This study broadens current knowledge of ARID1B mutations for clinicians and genetic consultors.

## Background

Coffin–Siris syndrome (CSS, OMIM#135900) is a rare congenital anomaly syndrome characterized by intellectual disability, growth deficiency, microcephaly, coarse facial features and hypoplastic nail of the fifth finger and/or toe [1]. Most of the cases were sporadic and showed an autosomal dominant mode of inheritance. The global prevalence of this disease was estimated at approximately 1:10,000–1:100,000 [2].

According to the available reports, the genetics of CSS is quite heterogenous. Currently, there are 12 types of CSSs caused by pathogenic mutations in different genes: CSS1 (OMIM#135900) by *ARID1B* (OMIM#614556) [2], CSS2 (OMIM#614607) by *ARID1A* (OMIM#603024) [3], CSS3 (OMIM#614608) by *SMARCB1* (OMIM#601607) [4], CSS4 (OMIM#614609) by *SMARCA4* (OMIM#603254) [4], CSS5 (OMIM#616938) by *SMARCE1* (OMIM#603111) [4, 5], CSS6 (OMIM#617808) by *ARID2* (OMIM#609539) [6], CSS7 (OMIM#618027) by *DPF2* (OMIM#601671) [7], CSS8 (OMIM#618362) by *SMARCC2* (OMIM#601734) [8], CSS9 (OMIM#615866) by *SOX11* (OMIM#600898) [9], CSS10 (OMIM#618506) by *SOX4* (OMIM#184430) [10], CSS11 (OMIM#618779) by *SMARCD1* (OMIM#601735) [11], and CSS12 (OMIM#619325) by *BICRA* (OMIM#605690) [12]. It is worth noting that mutations in another gene, *SMARCA2* (OMIM#600014) could lead to Nicolaides–Baraitser syndrome (NCBRS; OMIM#601358), which possessed similar phenotypes with Coffin–Siris syndrome [13–15]. Therefore, it is a great challenge for clinicians to identify the disease types and genetic inheritable patterns. With the application of candidate gene panels and whole exome sequencing (WES), the diagnostic yields have been improved greatly from about 15–20% with chromosomal microarray (CMA) to 35–50% of cases [16–19].

Here, through array-CGH and whole-exome sequencing (WES) techniques, we identified one 2.84 Mbp 6q25 microdeletion in case 1, two loss-of-function (LoF) variants in *ARID1B* (AT-rich interaction domain 1B) in case 2 (c.2332 + 1G > A) and case 3 (c.4741C > T, p.Q1581X) All of the three abnormalities were novel, not inherited from any of their parents. Because more than 10 types of Coffin-Siris syndrome, and many hospitalized patients with intellectual disability (ID) were in their childhood without distinct clinical phenotypes, it is difficult to identify the underlying genetic factors. The combination of array-CGH and WES might be an efficient methodology to pinpoint the causal mutations.

## Methods

### Sample collection

This study was conducted in accordance with the Code of Ethics of the World Medical Association (Declaration of Helsinki) for experiments involving humans. This study was approved by the Ethical Committee of the Shenzhen Bao’an Women’s and Children’s Hospital. Written informed consent was obtained from each individual. The clinical phenotypes were compiled in Table 1.

**Table 1:** Clinical information of the three cases

	Case 1 (Family1)	Case 2 (Family 2)	Case 3 (Family 3)
*Genetic detection*			
ARID1B mutations	−	c.2332 + 1G > A (splicing)	c.4741C > T (p.Q1581X)
arrayCGH	6q25.3 deletion	−	−
Cytogenetic band deleted	46,XX	46,XX	46,XX
*General information*			
Age at report	3y5m	2y11m	3y
Sex	Female	Female	Female
Birth weight (g)	2950	2650	3200
Birth height (cm)	NA	48	50
Head circumstances (cm)	NA	32	34
*Facial features*			
Thick hair	+	+	+
Thick eyebrows	+	+	+
Thick eyelashes	+	+	+
Orbital hypertelorism	−	+	−
Down-slanting palpebral fissure	−	+	+
Up-slanting palpebral fissure	−	−	−
Nasal root abnormality	−	+	−
Low set ears	−	−	+
Abnormal ears	−	−	−
Midface hypoplasia	+	+	+
Wide mouth	+	+	+
Long philtrum	−	−	−
Upper lip vermilion feature	−	+	+
Thick lower lip vermilion	−	+	+
Palatal abnormality	−	−	−
*Skeletal–limb*			
Transverse crease	+	+	+
Clinodactyly	+	−	−
Hypoplastic/absent fifth finger/toe	+	+	−
Hypoplastic/absent nail (fifth finger/toe)	+	+	−
Hypoplastic/absent nail (other fingers/toes)	−	−	−
Broad thumb	−	+	+
Prominent interphalangeal joints	−	−	−
Prominent distal phalanges	−	−	−
Scoliosis/spinal abnormalities	−	+	−
Joint laxity	+	+	+
*Nervous system*			
Developmental delay	+	+	+
Seizures	−	+	−
Speech delay	+	+	+
Structural brain abnormalities	−	−	−
Agenesis of corpus callosum	−	−	−
Hypotonia	+	+	+
Hypertonia	−	−	−
Abnormal shape of head	+	+	+
Growth restriction	+	+	+
Microcephaly	−	−	−
Others			
Hirsutism	−	+	+
Congenital heart defects	−	−	−
Genitourinary defects	−	−	−
Gastrointestinal abnormalities	−	−	−
Sucking difficulty	+	−	+
Feeding difficulty	−	+	+
Frequent vomiting	−	−	−
Hearing impairment	−	+	−
Visual impairment	−	−	−
Recurrent infections	−	−	−
*Family information*			
Siblings	One sister; normal	One brother; with attention deficit hyperactivity disorder	One brother; normal
Family history	No	No	No
Consanguineous marriage	No	No	No

Peripheral venous blood was collected from the three patients and their parents. Genomic DNA was extracted using the TIANamp Blood DNA Kit (DP348, Tiangen Biotech, Beijing, China) according to the manufacturer's instructions.

### Array-comparative genomic hybridization (Array-CGH)

Array-CGH was performed using the Fetal DNA Chip (Version 1.2) designed by The Chinese University of Hong Kong (CUHK) [20, 21]. The chip contains a total of 60,000 probes for more than 100 diseases caused by known microduplication/microdeletions. It doesn’t include small-size chromosomal abnormalities, copy number polymorphism, chimerism and chromosomal rearrangement [22]. The experimental procedures were performed according to the standard Agilent protocol (Agilent Oligonucleotide Array-Based CGH for Genomic DNA Analysis, version 3.5). Hybridized slides were scanned with SureScan High-Resolution Microarray Scanner (G2505B, Agilent Technologies, Santa Clara, CA, USA), and the image data were extracted and converted to text files using Agilent Feature Extraction software (Version 10.5.1.1). The data were graphed and analyzed using Agilent CGH Analytics software.

Only gains or losses that encompassed by at least three consecutive oligomers on the array were considered. Then, the clinical relevance of observed chromosomal aberrations was estimated according to data found in the scientific literature and databases for each of the regions and genes involved, using the DECIPHER database for known microdeletion and microduplication syndromes and the Online Mendelian Inheritance in Man (OMIM) for known disease-causing genes, gene functions, and inheritance patterns. DNA copy alterations were considered possibly pathogenic when they involved regions known to be associated with microdeletion or microduplication syndromes.

### Whole exome sequencing for the affected trios

To investigate the genetic cause of the disease, WES was performed for the trios of the two affected probands (case 2 and 3) at MyGenostics Co. LTD. Briefly, genomic DNA of each sample was quantified by NanoDrop spectrophotometry 8000 (Thermo Scientific, Waltham, MA, USA). 1 μg of genomic DNA was sheared by nebulization. Sheared DNA were ligated to the 3ʹ end of Illumina adapters. Products with 350–400 bp were amplified by polymerase chain reaction (PCR). The quality of the amplified products was checked using the Agilent Bioanalyzer (Agilent Technologies, Santa Clara, CA, USA). The amplified DNA was captured with Gencap Human whole Exon Kit (52 M) based on MyGenostics GenCap™ Enrichment Technologies (MyGenostics, Beijing, China). The capture procedure was performed according to the manufacturer’s protocol. Finally, the resultant libraries were sequenced on Illumina HiSeq 2500 platform for paired-end sequencing.

The sequencing depth was about 100× for each sample. Sequences were aligned to the human reference genome (UCSC hg19). ANNOVAR was applied to annotate the VCF file. Variants with a Minor Allele Frequency (MAF) > 0.1% or synonymous single nucleotide variants (SNVs) were removed. SNVs causing splicing, frameshift, stop gain or stop loss were kept for subsequent analysis. The location, type, conservation of the identified mutations was obtained from several public databases, such as UCSC Genome Browser, NCBI dbSNP, NCBI ClinVar, 1000 Genome and ExAC. The pathogenicity of the variants was evaluated according to the American College of Medical Genetics and Genomics (ACMG) guidelines [23] and the online software, PolyPhen-2 and SIFT for functional prediction. A position was called as heterozygous if 25% or more of the reads identify the minor allele.

### Protein interaction analysis

The 12 genes for CSS and 1 for NCBRS listed in OMIM were used as input to STRING Protein–Protein interaction database (http://string-db.org/), that holds experimental, predicted and transferred interactions together with interactions obtained through text mining [24]. To select stronger interactors, network clustering was performed using k-means algorithm (number of clustering was set as 4).

## Results

### Clinical and demographic characteristics of three cases

#### Case 1 (Family 1)

**Fig. 1:**
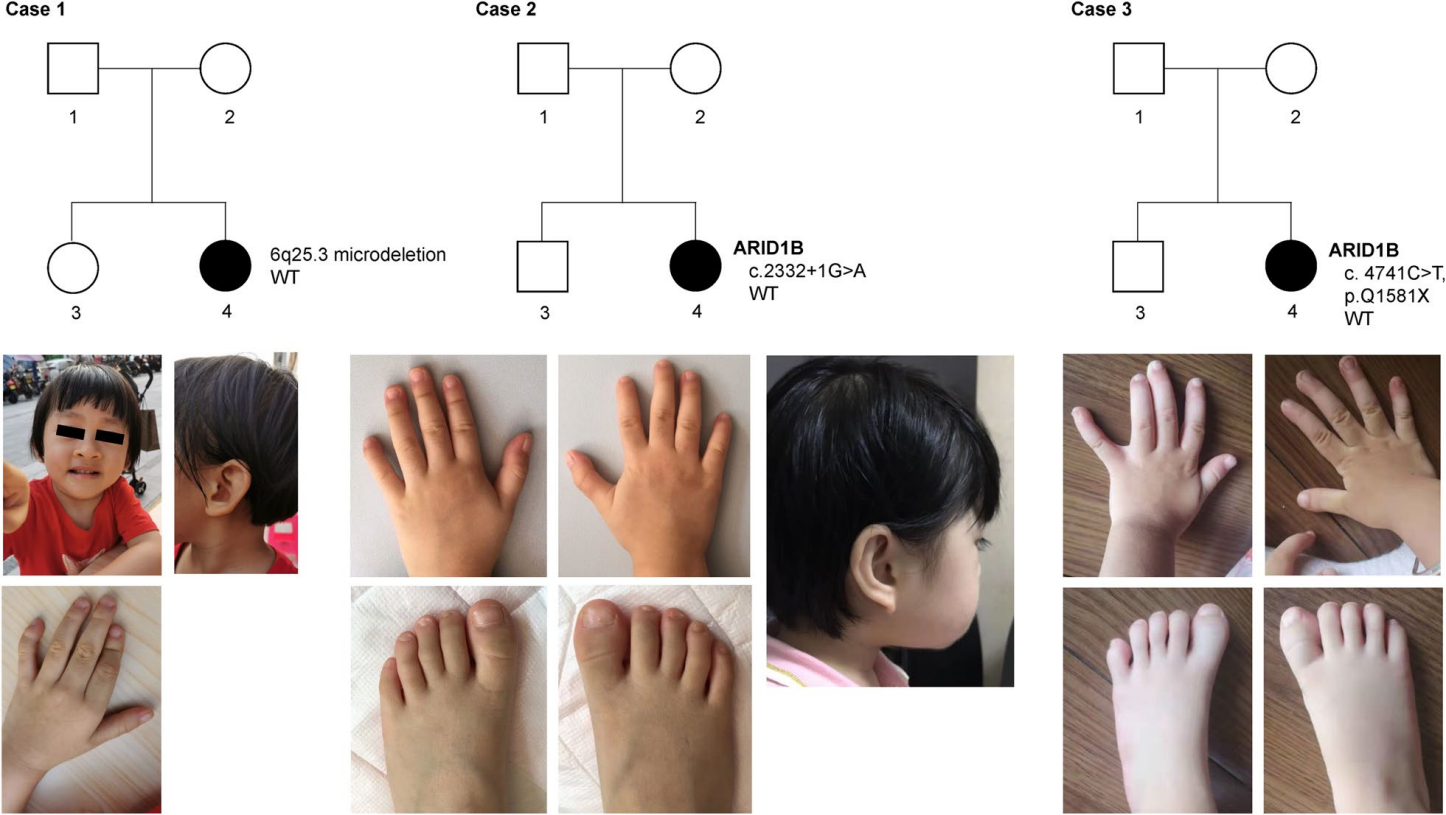
Pedigrees of the three patients. **a** Trio of case 1 with 6q25.3 microdeletion; **b** pictures of face and hand of case 1. **c** Trio of case 2 with c.2332 + 1G > A mutation in ARID1B; **d** Pictures of hands and foot of case 2; **e** trio of case 3 with c.4741C > T (p.Q1581X) mutation in ARID1B; **f** pictures of hands and foot of case 3

The girl was G2P2, born by cesarean section at full-term of pregnancy. The birth weight was 2.95 kg. At 1 year 6 months old, the child was unbale to speak, but not taken to seek medical advice. It was at the age of nearly 2 that the child could unconsciously made a "Baba-Mama" sound. At 2 years 4 months old, she was still unable to walk without support. Now, she was 3 years and 5 months old, with a weight at 11.9 kg, height 88 cm, and head circumstance 48.5 cm. The pedigree and some features were depicted in Fig. 1a and b. Family history: Her parents were healthy and non-consanguineous. Her mother was 30 years old and had a healthy living style during pregnancy. The patient has a healthy 6-year-old sister.

#### Case 2 (Family 2)

The girl was G5P2A3, born by cesarean section at 39 + 3 weeks of gestation, with a birth weight of 2.65 kg, birth height 48 cm and head circumstance 32 cm. Two months after birth, she was admitted to the Shenzhen Hospital affiliated to the University of Hong Kong for treatment due to five times of "suspicious convulsions " and diagnosed as "epilepsy". At 5 months old, her height increased to 90 cm, and weight to 11.6 kg. Her psychomotor development was significantly behind the children of the same age, and also combined with hypotonia. The pedigree and some features were depicted in Fig. 1c and d. Family history: Her healthy parents were not consanguineous. Her mother was 38 years old and had no history of smoking, drinking, long-term exposure to chemicals and harmful radiation during pregnancy. The patient has a 12-year-old brother suffered from "attention deficit hyperactivity disorder".

#### Case 3 (Family 3)

The girl was G2P2, born naturally at 39 + 1 weeks of gestation with a birth weight of 3.2 kg, height 50 cm and head circumstance 34 cm. Her crying voice was weak at birth. She could not take the initiative to suck in the first two months after birth and was fed with dropper. On the 4th day after birth, she was hospitalized for 1 week due to "neonatal hyperbilirubinemia". About 1 year ago (at 8 months and 21 days old), the child could not sit alone and was treated as "developmental delay". Her cognitive and motor development was significantly lagging behind children of the same age. She could understand and execute simple instructions, and uttered no more than 5 words. At this time, her height was 94 cm, with a weight at 12.4 kg. The pedigree and some features were depicted in Fig. 1e and f. Family history: She was born of healthy, non-consanguineous parents. Her mother was 35 years old and had no history of smoking, drinking, long-term exposure to chemicals and harmful radiation during pregnancy. The patient has a healthy brother.

Clinical characters of the facial, skeletal–limb, nervous system and other features of the three cases were compiled in Table 1.

### A 6q25 microdeletion was identified in case 1 by array-CGH

**Fig. 2:**
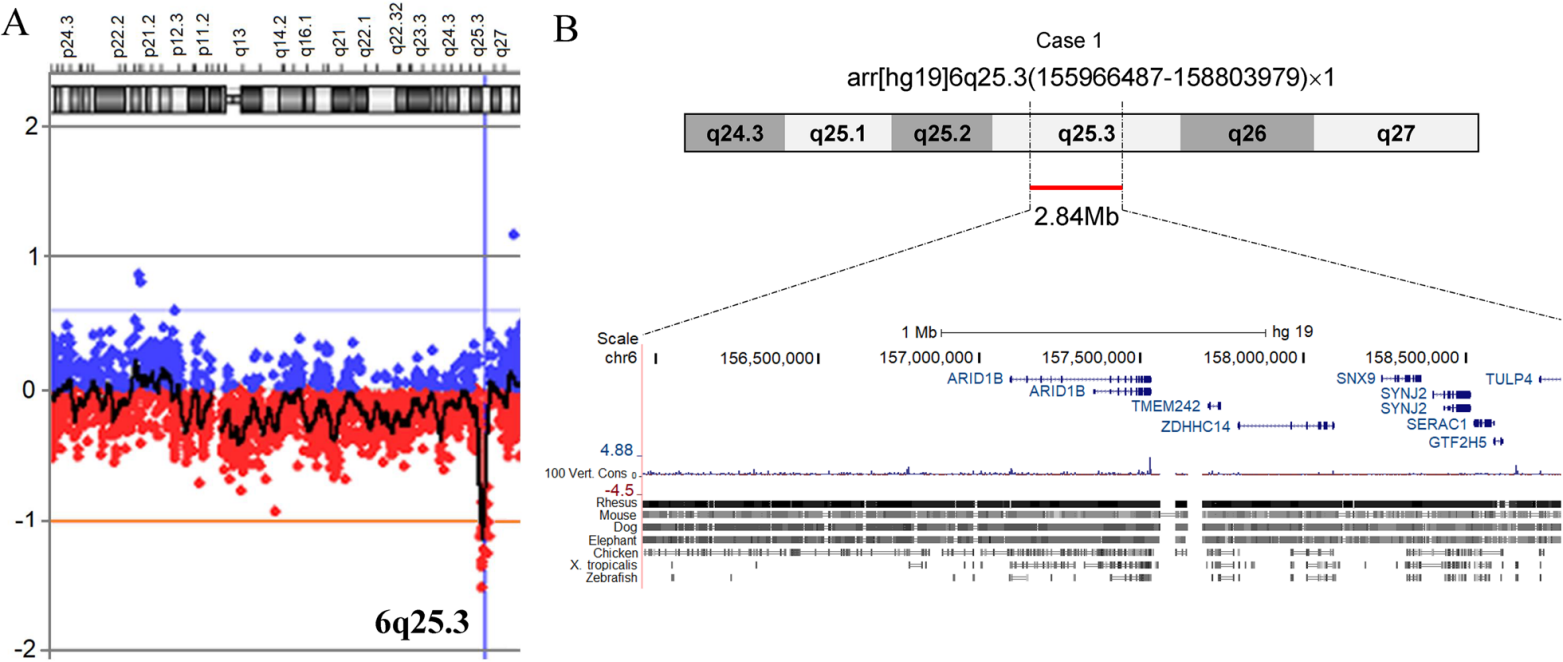
A 6q25.3 microdeletion detected in Case 1. **a** 6q25.3 microdeletion was identified by array-CGH. **b** Seven protein-coding genes including ARID1B in the microdeletion region

Oligonucleotide array-CGH was performed for the three patients using the Fetal DNA Chip (Version 1.2) designed by the Chinese University of Hong Kong (CUHK). A microdeletion at 6q25.3 was detected in case 1, arr[hg19]6q25.3(155,966,487–158,803,979) × 1. The length of this microdeletion was about 2.84 Mbp (chr6:155,966,487–158,803,979) (Fig. 2a). This deletion was not identified in her parents. Besides, there were no deletions or duplications detected in case 2 and case 3.

This deleted region contains 7 protein-encoding genes, and is highly conserved in mammals (Fig. 2b). Five of them (*ARID1B, ZDHHC14, SNX9, SYNJ2* and *GTF2H5*) were localized on the sense strand and two (*TMEM242* and *SERAC1*) on the antisense strand.

### Two novel pathological point mutations were identified by WES

As for the other two cases, WES was performed at MyGenostics (MyGenostics, Beijing, China). The aligned bases for case 2 and case 3 were 13,859.9 and 15,678.15 Mb, respectively. The ratios of the coverage on target regions were 99.33% and 99.69%, respectively. The average sequencing depths on target regions were 109.65 and 118.64 for case 2 and case 3, respectively.

**Fig. 3:**
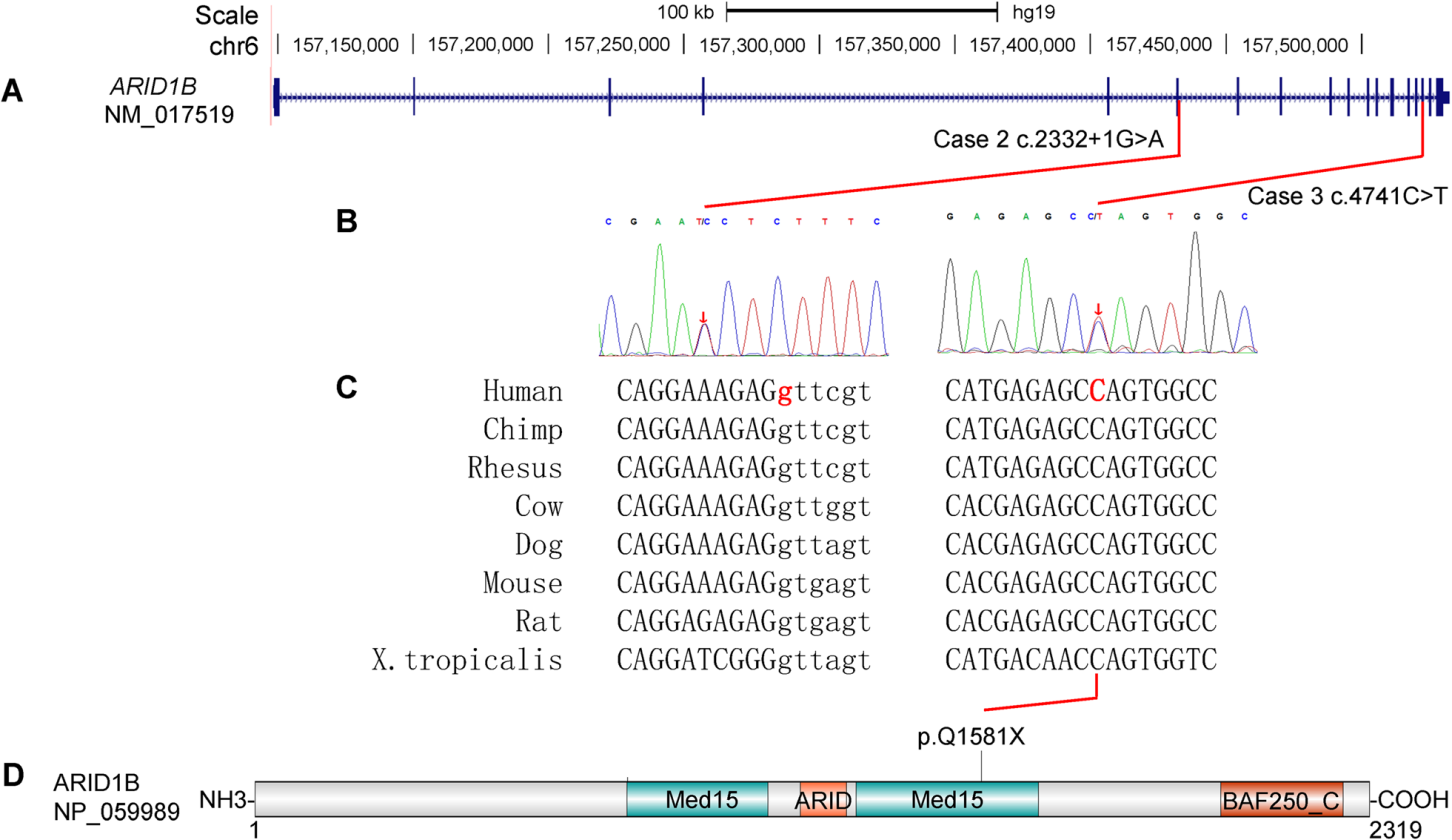
Two de novo mutations of ARID1B gene in case 2 and case 3. **a** Genomic structure of ARID1B gene; Thin box represents exons, line represents introns. **b** Sanger sequencing of the two single-nucleotide mutations. **c** Conservational analysis of the sequences around the two mutations in different organisms. **d** Location of the p.Q1581X in the protein sequence of ARID1B

In case 2, a heterozygous SNV was detected at the splicing donor site of exon 6 (c.2332 + 1G > A, chr6:157,431,696) of *ARID1B* (NM_017519) (Fig. 3a, Table 2). It had been confirmed by Sanger sequencing (Fig. 3b). After comparing the sequences from human, chimpanzee, rhesus, cow, dog, mouse, rat and X. tropicalis, this nucleotide (2332 + 1G) was strongly conserved during evolution. This SNV was not detected in the several public genomic databases, such as 1000Genome Project (n = 2504), NHLBI Exome Sequencing Project (GO-ESP) (n = 6503), The Exome Aggregation Consortium (ExAC) (n = 60,706), gnomAD (n = 15,708) and NHLBI Trans-Omics for Precision Medicine (TOPMED) (n = 60,000). Besides, this mutation was not identified in the parents’ genomes. Therefore, this mutation was appeared spontaneously in case 2 and should be regarded as ‘de novo’. According to criteria of ACMG guidelines, this SNV was classified as PVS1 + PS2 + PM2 and annotated as “Likely pathogenic”.

**Table 2:** De novo mutations of ARID1B identified in two cases

Cases	Sex	Location (GRCh37/hg19)	Nucleotide	Amino acid	Zygosity	dbSNP ID	Origin	ACMG	Population frequencies	Impact
2	Female	chr6:157,431,696	c.2332 + 1G > A	–	Het	–	De novo	PVS1 + PS2 + PM2	–	Splicing donor loss
3	Female	chr6:157,522,259	c.4741C > T	p.Q1581X	Het	rs1554235831	De novo	PVS1 + PS2 + PM2	–	Stop gain

Het, heterozygous; X, stop codon; PVS1, pathogenic very strong, represent “loss-of-function”; PS2, pathogenic strong, 2 represent “de novo”; PM2; pathogenic moderate, 2 represent “absent from controls”

In case 3, a nonsense mutation (c.4741C > T, chr6:157,522,259) was identified in the exon 18 of *ARID1B* (NM_017519), causing the codon (CAG) for Gln (Q) to be a premature stop codon (TAG, X) (p.Q1581X) (Fig. 3a, d, Table 2). This mutation was verified by Sanger sequencing (Fig. 3c). This nucleotide (4492C) was strongly conserved during evolution. The mutation was not included in the large-scale genomic databases mentioned above. Since this stop gain mutation was only identified in case 3 and not in her parents, it was regarded as another ‘de novo’ variant. Besides, the variant has been annotated as rs1554235831 in NCBI dbSNP database. According to the criteria of ACMG guidelines, p.Q1581X was classified as PVS1 + PS2 + PM2 and annotated as “pathogenic”.

### 6q25 Microdeletions involving ARID1B

**Fig. 4:**
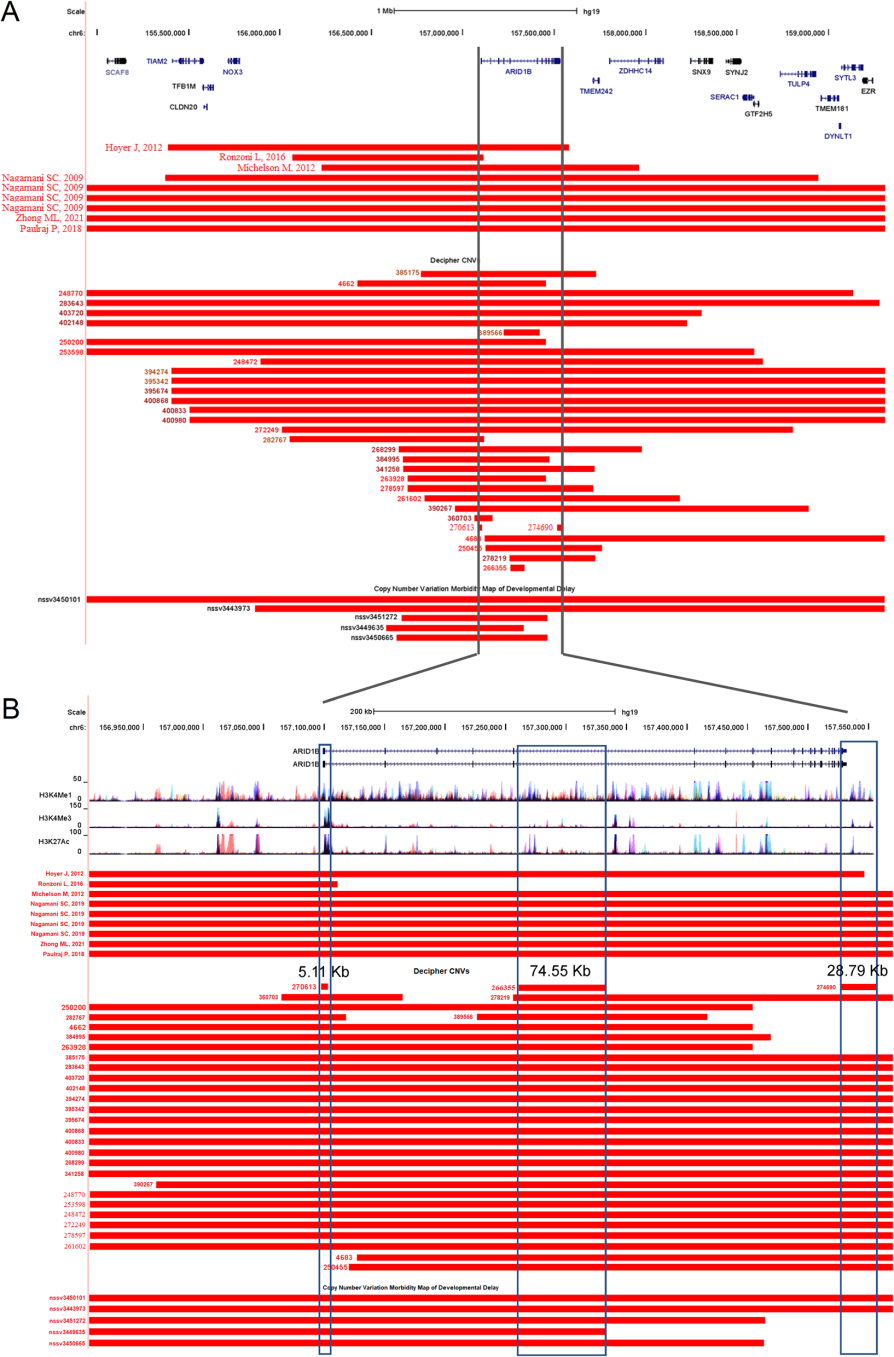
Mapping of microdeletions involving ARID1B. **a** Mapping of the microdeletions involving ARID1B. **b** Zoomed-in view of the microdeletions. Red bar represents microdeletions

9 reported 6q25 microdeletions associated with *ARID1B*-related disorders were recruited from published articles. We also collected individuals with developmental disorders whose genomes containing microdeletions involving *ARID1B* gene from DECIPHER [25, 26] and Developmental Delay [27, 28]. 32 were from DECIPHER and 5 from Developmental Delay. The microdeletions were compared against human genome (hg19) using UCSC Genome Browser. Totally, 46 microdeletions were recruited for the subsequent analysis (Fig. 4a). 39 of them were completely (or almost) covered the whole genomic region of *ARID1B*. As for the remaining partially-covered microdeletions, the shortest one (270,613, chr6:157,096,761–157,101,867) was only 5.11 Kb, just spanning the promoter region of *ARID1B*, just as the deletions reported by Ronzoni L in 2016 and detected in two DECIPHER samples (282,767 and 360,703). There also existed a 28.79 Kb microdeletion (274,690, chr6:157,527,273–157,556,065), which covered the whole last exon of *ARID1B*, thus removing part of the protein coding sequence and the complete 3’-untranslated region (3’-UTR) of *ARID1B*. Interestingly, the microdeletion (266,355, chr6:157,261,502–157,336,055) located in the fifth intron and did not affect the protein coding region of *ARID1B*. According to the chromatin modification patterns from 7 cell lines (GM12878, H1-hESC, HSMM, HUVEC, K562, NHEK and NHLF) from ENCODE (Encyclopedia of DNA Elements) [29], several candidate enhancers were located in the region covered by this deletion (266,355) (Fig. 4b).

## Discussion

**Fig. 5:**
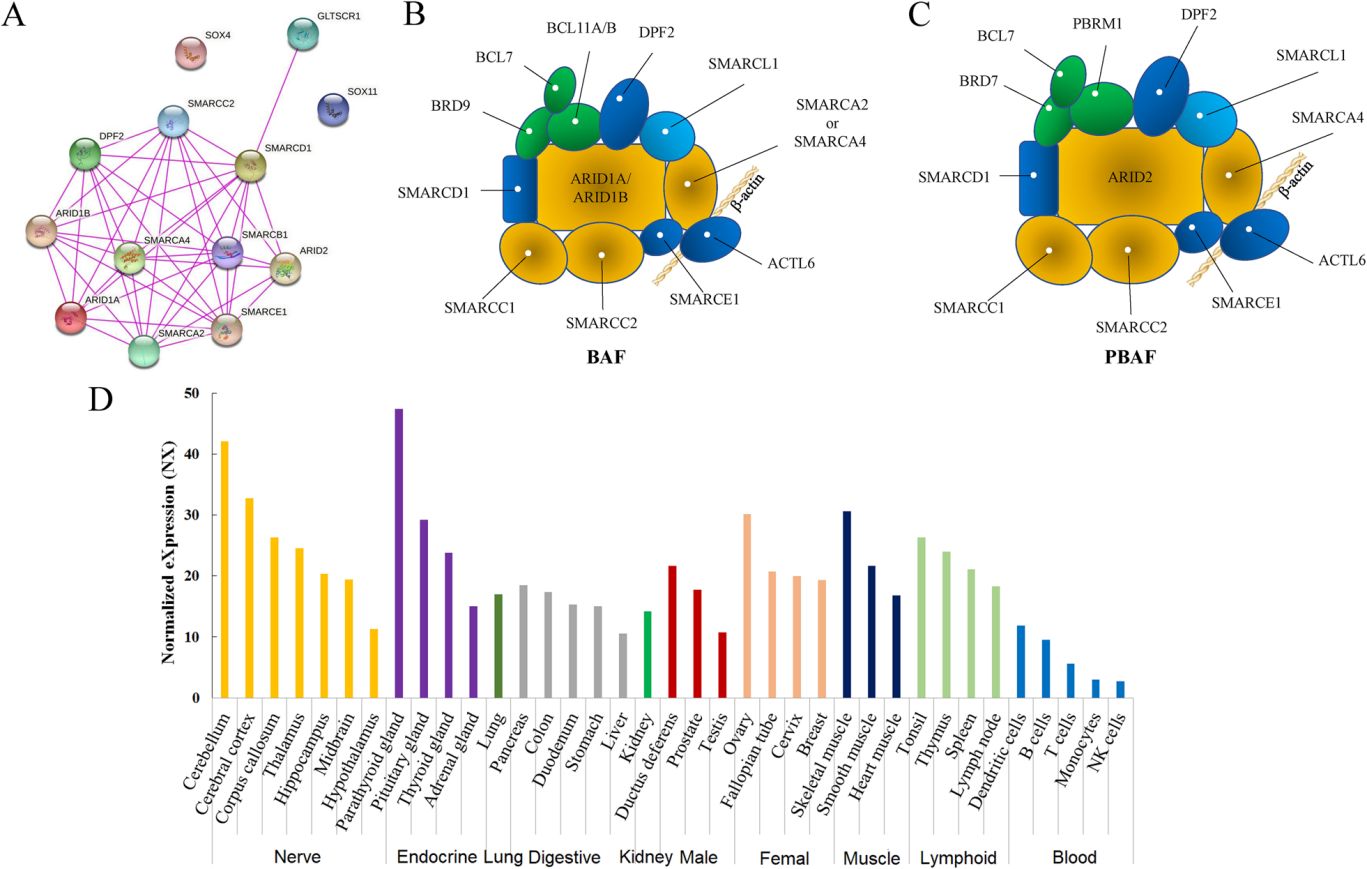
Interaction of proteins for CSS and structures of SWI/SNF-related proteins. **a** Interactions of the 12 proteins for Coffin–Siris syndrome and 1 for NCBRS. **b** Structures of BAF. **c** Structures of PBAF. **d** Expression of ARID1B in different tissues of human (adopted from the Human Protein Atlas)

Currently, there are 12 genes responsible for CSSs and 1 for NCBRS, a disorder with similar phenotypes of CSSs. Among the 13 genes, except the two transcription factors, SOX4 and SOX11, proteins encoded by the remaining 11 genes were bound with each other (Fig. 5a) to form two SWI/SNF-related complex, BAF (Brg/Brahma-associated factors) complex and/or PBAF (Polybromo BRG1 Associated Factor) (Fig. 5b, c). The SWI/SNF complex was originally referred to as the protein complex critical for cellular responses to mating-type switching (SWI) or sucrose fermentation (SNF) in yeast [30, 31]. This multi-protein complex contains more than 15 subunits to activate gene expression through its capacity to remodel and remove nucleosomes at gene promoters [32]. Recently, mutations, translocations and deletions of the subunits in the SWI/SNF complex have been linked to a number of human diseases, such as cancer [33], different types of CSS [4, 34–38] and NCBRS [15].

The gene for CSS1 is *ARID1B* [2, 4, 39], a core subunit of the BAF complex. This gene is the most frequently mutated genes in cases with CSS [5]. The phenotypes caused by *ARID1B* mutations encompass a spectrum of features, including feeding difficulties, laryngomalacia, speech delay, motor delay, hypertrichosis, and cryptorchidism [40]. According to the Human Protein Atlas, ARID1B is expressed ubiquitously, and abundantly detected in never, endocrine, muscle and lymphoid systems (Fig. 5d). It is reported that meticulous coordination between the actin cytoskeleton and the microtubule network regulate the formation and transportation of secretory vesicles for proper neurite outgrowth and maintenance, which is critical for normal neural development [41]. This finely-tuned coordination is regulated by the BAF complex [42]. ARID1B (previously named as BAF250B) is a core member of the BAF complex and plays an essential role for dendrite outgrowth and arborization in cortical and hippocampal pyramidal neurons during brain development in mice [43]. ARID1B deficiency led to decreased dendritic branching, thus hinder the dendritic innervation into cortical layer I to form proper synapses. This might disrupt the balanced excitatory and inhibitory inputs and result in pathologic phenotypes of ID.

### ARID1B is the crucial pathogenic factor behind 6q25 microdeletion-related disorder

In our case 1, there exists a heterozygous microdeletion arr[hg19]6q25.3(155,966,487–158,803,979) × 1 (Fig. 3a). This region contains 7 protein-coding genes, namely, *ARID1B, TMEM242, ZDHHC14, SNX9, SYNJ2, SERAC1* and *GTF2H5*. It is worth noting that several inheritable disease-causing genes were recruited in the OMIM database. *ARID1B* (OMIM#614556) is the causal gene for CSS1 (OMIM#135900). *GTF2H5* (general transcription factor IIH subunit 5, OMIM#608780) could result in the photosensitive trichothiodystrophy-3 (TTD3) (OMIM#616395) [44–46], a rare autosomal recessive disorder characterized by brittle sulfur-deficient hair, ichthyosis, developmental disabilities, decreased fertility, ocular abnormalities, short stature, and infections [47]. *SERAC1* (serine active site containing 1, OMIM#614725) functions in phosphatidylglycerol remodeling that is essential for mitochondrial function and intracellular cholesterol trafficking [48]. Bi-allelic Mutations of this gene were associated with 3-methyl-glutaric aciduria, accompanied with deafness, encephalopathy and Leigh-like syndrome (MEGDHEL, OMIM#614739) [48–50]. *TMEM242* was reported to affect the assembly of the ATP synthase and mitochondrial complex I, to a certain degree [51]. *ZDHHC14* (zinc finger DHHC-type palmitoyl transferase 14) is highly expressed in the hippocampus and is the only palmitoyl acyltransferase (PAT) predicted to bind Type-I PDZ domain-containing Membrane-associated Guanylate Kinase PSD93, which localizes to the axon initial segment (AIS) [52]. Loss of *ZDHHC14* decreases outward currents and increases action potential firing in hippocampal neurons. SNX9 (Sorting nexin 9), a member of the sorting nexin family, is required for membrane remodeling during endocytosis [53]. As for *SYNJ2* (synaptojanin 2), it is a ubiquitously expressed phosphoinositol 5-phosphatase, involved in vesicular trafficking and actin dynamics. Although it is not clear whether SYNJ2 played a role in the normal brain development, an association study showed that SYNJ2 was associated with general memory and general cognitive ability [54].

Based on the above analysis, *ARID1B, GTF2H5, SERAC1* and *ZDHHC14* might be the promising candidates responsible for the 6q25 microdeletion syndrome. Currently, there are more than 10 reported individuals harboring 6q25 microdeletion [2, 55–59]. The reported shortest 6q25 deletion (1.1 Mb, chr6:156,004,307–157,120,089) contained only one protein-coding gene, *ARID1B* [60]. According to the ARID1B-related deletions collected in DECIPHER and Developmental Delay, the shortest deletion (270,613) was only 5.11 Kb, just spanning the promoter region and first exon of ARID1B. This deletion made *ARID1B* unable to start the transcription, just as the deletions reported by Ronzoni L in 2016 and detected in two DECIPHER samples (282,767 and 360,703). There also existed a 28.79 Kb microdeletion (274,690), which covered the whole last exon of *ARID1B*, thus removing part of the protein-coding sequence and the complete 3′-UTR of *ARID1B*. The transcription of ARID1B could not stop at the normal termination site, and might produce truncated proteins without the C-terminal BAF250_C domain (pfam12031). Interestingly, the microdeletion (266,355) just located in the middle of *ARID1B*, just like the sample 389,566. According to the chromatin modification patterns from 7 cell lines (GM12878, H1-hESC, HSMM, HUVEC, K562, NHEK and NHLF) from ENCODE, several candidate enhancers were located in this region (Fig. 5b). Loss of these enhancers might affect the transcription efficiency of *ARID1B*. Therefore, the insufficient production of ARID1B could be caused by four types of 6q25 microdeletions, namely whole genomic region, promoter region, termination region and enhancer regions.

Haploinsufficiency of *ARID1B* has been reported to be recurrently detected in intellectual disability (ID) or mental retardation (MRT) [60, 61], therefore, *ARID1B* might be the crucial pathogenic factor behind the 6q25 microdeletion syndrome.

### Haploinsufficiency of ARID1B caused by de novo SNVs

In case 2, a variant at the splicing site of exon 6 (c.2332 + 1G > A) of *ARID1B* (NM_017519) was identified. This variant disrupted the splicing donor site (GT) of intron 6, which might affect the proper splicing of *ARID1B*’s mRNA during transcription to produce abnormal transcripts, thus to inactivate the function of ARID1B. It is reported that heterozygous mutations of splicing sites could affect splicing and lead to haploinsufficiency of the affected genes [62, 63]. Therefore, it is reasonable that c.2332 + 1G > A mutation might be detrimental the proper splicing and result to insufficient expression of *ARID1B*.

In case 3, the mutation (c.4741C > T) was located in exon 18 of ARID1B, causing the codon (CAG) for Q to be a premature termination codon (TAG) (p.Q1581X). The transcript contains two stop codons (one at position 1581 and another at position 2237). It had been reported that mRNAs containing premature termination codons (PTC) could be detected and degrade rapidly by a special mRNA surveillance mechanism, Nonsense-mediated mRNA decay (NMD). It is widely accepted that the biological purpose of NMD is to protect cells from potential harmful effects caused by truncated translational products as a consequence of frameshift or nonsense mutations or by inaccurate pre-mRNA splicing [64–66]. It is been found that in 143 patients with CSS, all pathogenic variants were truncating (nonsense, frameshift, splice-site, and deletions of various numbers of exons including whole-gene deletions). Therefore, these two de novo single nucleotide variants in our project might lead to haploinsufficiency of ARID1B and be pathogenic for CSS.

### Treatments for ARID1B-related disorders

Till now, there has no effective treatments for ARID1B-related disorders. The present methods are symptomatic treatment and cannot cure this disease. Currently, a clinical trial is ongoing to investigate the effects of clonazepam on children with ARID1B-related intellectual disability in Netherlands (EudraCT: 2019-003558-98). The purpose of this clinical trial is to test the beneficial effects of clonazepam on behavior and cognitive function in ARID1B patients.

Animal with ARID1B haploinsufficiency (Arid1b^+/−^) would be promising models for the study of molecular mechanism and discovering of drugs for ARID1B-related disorder. The Arid1b^+/−^ C57BL/6J mice showed reduced corpus callosum size, dentate gyrus size, cortex thickness, and proliferation [67–69]. It was found that deficiency of GHRH–GH–IGF1 axis was detected in Arid1b^+/−^ mice [67]. Exogenous GH supplementation could significantly reverse the growth retardation in Arid1b^+/−^ mice, but no improvement on abnormal behavioral phenotypes such as anxiety. This indicated there might be other critical unknown druggable targets for the treatment of ARID1B-related disorder. Or Arid1b^+/−^ mice might not be the most suitable animal for the study of ARID1B-related disorder.

## Conclusions

Here we identified a patient with haploinsufficiency of ARID1B caused by a 2.84 mb 6q25 microdeletion and two caused by loss-of-function (LoF) mutations of *ARID1B*. All of the three abnormalities were acquired spontaneously. *ARID1B* gene was the critical genetic factor for ARID1B-related disorder. Besides, four types of 6q25 microdeletion were identified by silico analysis. This would broaden the knowledge about *ARID1B* mutation spectrum for clinicians and genetic counselor.

## Data Availability

The datasets about whole exome sequencing used for the current study were available at https://www.ncbi.nlm.nih.gov/sra/?term=PRJNA777349.

## Abbreviations

CSS: Coffin–Siris syndrome
LoF: Loss-of-function
NCBRS: Nicolaides–Baraitser syndrome
SWI/SNF: Mating-type switching (SWI) or sucrose fermentation (SNF)
ARID1B: AT-rich interaction domain 1B
Array-CGH: Array-comparative genomic hybridization
CUHK: Chinese University of Hong Kong
MAF: Minor allele frequency
OMIM: Online Mendelian inheritance in man
WES: Whole-exome sequencing
SNVs: Single nucleotide variants
ExAC: The exome aggregation consortium
ESP: NHLBI exome sequencing project
GTF2H5: General transcription factor IIH subunit 5
TTD3: Trichothiodystrophy-3
SERAC1: Serine active site containing 1
MEGDHEL: 3-Methyl-glutaric aciduria, accompanied with deafness, encephalopathy and Leigh-like syndrome
ZDHHC14: Zinc finger DHHC-type palmitoyl transferase 14
PAT: Palmitoyl acyltransferase
AIS: Axon initial segment
SNX9: Sorting nexin 9
SYNJ2: Synaptojanin 2
ID: Intellectual disability
MRT: Mental retardation
PTC: Premature termination codons
NMD: Nonsense-mediated mRNA decay
